# Remimazolam is associated with superior cerebral and pulmonary protection over propofol in elderly thoracic surgery: a real-world study validated by propensity score matching

**DOI:** 10.3389/fmed.2026.1805497

**Published:** 2026-06-03

**Authors:** Jingjing Zhang, Xiaoyan Zhang, Na Cao, Yichao Cai, Caixia Zhao, Chunying Tang, Zhenhua Ma, Yan Zhang

**Affiliations:** Department of Anesthesiology, Zhangjiakou First Hospital, Zhangjiakou, Hebei, China

**Keywords:** cerebral oximetry, elderly, postoperative cognitive dysfunction, postoperative pulmonary complications, propensity score matching, propofol, real-world evidence, remimazolam

## Abstract

**Background:**

Selecting the optimal anesthetic regimen to mitigate postoperative cognitive decline and pulmonary complications in elderly patients undergoing thoracic surgery remains a clinical challenge. While propofol is widely used, remimazolam offers potential advantages due to its hemodynamic stability and rapid clearance. This study employed rigorous propensity score matching (PSM) to compare the real-world outcomes of these two intravenous anesthetics in a high-risk surgical cohort.

**Methods:**

We conducted a retrospective cohort study of patients aged ≥65 years undergoing elective VATS lobectomy (2020–2023). Patients received either remimazolam- or propofol-based total intravenous anesthesia under a standardized, driving pressure-guided individualized PEEP protocol. To address significant baseline imbalances and confounding by indication, we performed 1:1 propensity score matching using a multivariable logistic regression model incorporating 13 pre-specified covariates (e.g., age, comorbidities, pulmonary/cognitive function). Balance was assessed via standardized mean differences. The matched cohort was used for all outcome analyses.

**Results:**

From 342 eligible patients, PSM created 122 well-balanced pairs (*n* = 244). The matched cohort demonstrated excellent covariate balance (all SMDs < 0.1). Compared to the propofol group, the remimazolam group exhibited: (1) Superior cognitive recovery across the early and short-term postoperative period, with higher MMSE scores at 24 h (mean difference 3.6 points, 95% CI 2.8–4.4, *p* < 0.001), 72 h (2.7 points, 95% CI 2.0–3.4, *p* < 0.001), postoperative day 4 (2.1 points, 95% CI 1.4–2.8, *p* < 0.001), day 7 (1.3 points, 95% CI 0.7–1.9, *p* < 0.001), and day 30 (0.6 points, 95% CI 0.1–1.1, *p* = 0.021); (2) Better pulmonary function at 72 h, including higher FEV₁% predicted (6.9%, 4.3–9.5, *p* < 0.001) and lower lung ultrasound scores (−2.0, −2.4 to −1.6, *p* < 0.001); (3) Enhanced hemodynamic and cerebral oxygen stability, with higher time-weighted mean arterial pressure (5.7 mmHg, 3.9–7.5, *p* < 0.001) and cerebral oxygen saturation nadir during one-lung ventilation (4.3%, 2.9–5.7, *p* < 0.001); and (4) Reduced postoperative morbidity, including lower incidence of delirium (9.0% vs. 23.0%, OR 0.33, 95% CI 0.16–0.69) and pulmonary complications (11.5% vs. 22.1%, OR 0.46, 0.23–0.92).

**Conclusion:**

Using robust propensity score matching to emulate a randomized comparison, this study provides real-world evidence that a remimazolam-based anesthetic strategy, integrated with lung-protective ventilation, is associated with significantly better cerebral oxygenation, accelerated neurocognitive and pulmonary recovery, and lower rates of key postoperative complications compared to propofol in elderly patients undergoing VATS lobectomy. These findings highlight the clinical value of remimazolam within enhanced recovery pathways for geriatric thoracic surgery.

## 1 Introduction

The global aging population has led to a marked increase in thoracic surgeries among elderly patients (1). Although video-assisted thoracoscopic surgery (VATS) reduces surgical trauma, older individuals remain vulnerable to perioperative complications, particularly postoperative cognitive dysfunction (POCD) and pulmonary morbidity (2, 3). Thoracic surgery with one-lung ventilation (OLV) imposes unique insults—hypoxemia, inflammation, and ventilator-induced lung injury—while hemodynamic shifts may compromise cerebral perfusion, a key contributor to POCD and postoperative delirium (POD) (4–6).

The choice of anesthetic agent is a modifiable factor affecting these risks. Propofol, widely used for total intravenous anesthesia (TIVA), causes dose-dependent cardiovascular depression and hypotension, which can reduce cerebral perfusion pressure (7, 8). Benzodiazepines like midazolam offer better hemodynamic stability but have prolonged context-sensitive half-lives and active metabolites, leading to delayed recovery in the elderly (9).

Remimazolam, an ultra-short-acting benzodiazepine, acts as a positive allosteric modulator of GABA_A receptors and is rapidly hydrolyzed by tissue esterases, yielding a predictable, rapid recovery independent of hepatic or renal function (10, 11). It appears to cause less vasodilation and myocardial depression than propofol, providing superior hemodynamic stability (12, 13). Recent meta-analyses confirm a lower risk of intraoperative hypotension with remimazolam in elderly patients (14). Concurrently, lung-protective ventilation with individualized PEEP is standard of care to mitigate postoperative pulmonary complications (15). The hemodynamic stability of remimazolam may facilitate consistent application of such PEEP levels without frequent downward titration due to hypotension. Recent RCTs have shown favorable hemodynamics and cognitive profiles with remimazolam compared to propofol during OLV (12), and attenuation of inflammatory responses and neurocognitive disorders in VATS patients (16). However, real-world data integrating detailed assessments of cerebral oxygenation, postoperative pulmonary function, and delirium under a standardized, individualized PEEP strategy remain scarce.

Therefore, this retrospective cohort study aimed to compare remimazolam-based versus propofol-based anesthesia—both administered under a uniform, driving pressure-guided individualized PEEP protocol—on a comprehensive set of outcomes in elderly patients undergoing VATS lobectomy. We hypothesized that, in a propensity score-matched cohort, remimazolam would be associated with greater hemodynamic stability, better-preserved cerebral oxygenation, enhanced neurocognitive and pulmonary recovery, and a lower incidence of postoperative delirium.

## 2 Methods

### 2.1 Study design, ethical framework, and data sources

This was a single-center, retrospective, propensity score-matched cohort study comparing remimazolam-based versus propofol-based general anesthesia—both administered under a uniform, individualized PEEP titration protocol—in elderly patients undergoing elective VATS lobectomy. The primary outcomes were early neurocognitive recovery and pulmonary function; secondary outcomes included hemodynamic stability, cerebral oxygenation, and incidence of postoperative delirium and pulmonary complications. The study protocol was approved by the Ethics Committee of Zhangjiakou First Hospital (2023-KY-48). Due to the retrospective, anonymized nature of the analysis, the requirement for written informed consent was waived.

Data were systematically extracted from three interoperable electronic databases: the Hospital Information System (demographics, ICD-10 diagnoses, laboratory results, medication records, nursing notes), the Anesthesia Information Management System (1-min interval vital signs, ventilator parameters, drug administration timing and doses, fluid balance), and the Surgical Procedure Database (resection type, operative duration, blood loss, pathology). Radiological studies were reviewed via PACS. Two independent reviewers extracted data; discrepancies were resolved by consensus or a senior investigator.

### 2.2 Study population, eligibility criteria, and data extraction process

We screened consecutive patients aged ≥65 years who underwent elective, curative-intent VATS lobectomy from January 2020 to December 2023. After an initial automated query (CPT 32663), two trained reviewers independently conducted a manual chart review using a standardized electronic case report form, which had been piloted on 20 non-study cases.

Inclusion criteria: (1) age 65–85 years; (2) ASA physical status I–III; (3) planned and completed elective VATS lobectomy for suspected primary lung cancer; (4) total intravenous anesthesia with remimazolam or propofol as the primary hypnotic for both induction and maintenance; (5) documented application of the institutional lung-protective ventilation protocol with individualized PEEP titration.

Exclusion criteria: emergency surgery; conversion to open thoracotomy; non-lobar resection (e.g., segmentectomy, wedge resection, pneumonectomy); pre-existing major neurocognitive disorder (e.g., dementia, Alzheimer’s disease) or severe psychiatric illness (e.g., schizophrenia); conditions impeding reliable cognitive testing (e.g., global aphasia, severe untreated hearing loss); severe baseline pulmonary dysfunction (FEV₁ < 50% predicted or chronic home oxygen therapy); Child-Pugh C hepatic insufficiency or end-stage renal disease requiring dialysis/transplantation; incomplete records on key anesthetic variables, PEEP titration, or primary outcomes.

After this rigorous process, 342 eligible patients formed the full unmatched cohort.

### 2.3 Anesthetic management, ventilation protocol, and standardized perioperative care pathways

All patients followed the institution’s Enhanced Recovery After Thoracic Surgery (ERATS) pathway. Premedication: intravenous midazolam 1–2 mg. Standard monitoring (ECG, pulse oximetry, NIBP, capnography) was applied; an arterial catheter was placed for continuous blood pressure monitoring and blood gas sampling. Depth of anesthesia was monitored with Bispectral Index (BIS, target 40–60).

The choice of anesthetic regimen was at the discretion of the attending anesthesiologist.

Remimazolam Group (Group R): Induction with remimazolam tosilate 0.2–0.3 mg/kg, sufentanil 0.3–0.5 μg/kg, rocuronium 0.6 mg/kg. Maintenance: continuous infusion of remimazolam 0.5–1.0 mg/kg/h plus remifentanil 0.05–0.20 μg/kg/min. Supplemental boluses of sufentanil (5–10 μg) and rocuronium (based on train-of-four monitoring) were given as needed.

Propofol Group (Group B): Induction with propofol 1.5–2.5 mg/kg, same doses of sufentanil and rocuronium. Maintenance: target-controlled infusion of propofol (plasma target 3–5 μg/mL) plus remifentanil 0.05–0.20 μg/kg/min.

In both groups, infusion rates were adjusted to maintain BIS 40–60 and systolic blood pressure within 20% of baseline. A left-sided double-lumen tube was placed and confirmed by fiberoptic bronchoscopy.

Lung-protective ventilation with individualized PEEP: After a recruitment maneuver (CPAP 30 cmH₂O for 30 s), volume-controlled ventilation was initiated with: tidal volume 6 mL/kg predicted body weight, FiO₂ 0.5, I: E 1:2, rate 12–14/min. PEEP was increased stepwise from 5 to 12 cmH₂O in 1-cmH₂O increments. At each PEEP level, after allowing five complete respiratory cycles for stabilization, an end-inspiratory pause of 0.5 s was applied to measure plateau pressure (Pplat). Driving pressure (ΔP = Pplat – PEEP) was calculated in real time. The PEEP that produced the lowest ΔP was selected; if two consecutive PEEP levels yielded the same minimal ΔP, the lower PEEP was chosen to minimize barotrauma and hemodynamic compromise. This individualized PEEP was maintained constant throughout both two-lung and one-lung ventilation. Ventilator adjustments were permitted only to keep PaCO₂ 35–45 mmHg, verified by intermittent arterial blood gas analysis.

Postoperative analgesia: ultrasound-guided thoracic paravertebral block (0.375% ropivacaine 20–30 mL) performed at the end of surgery, plus intravenous patient-controlled analgesia (PCA) with sufentanil (background infusion plus bolus) initiated in the post-anesthesia care unit.

### 2.4 Variable definitions, outcome measures, and data collection procedures

The primary exposure variable for this comparative effectiveness study was the type of intravenous hypnotic agent used for the maintenance of general anesthesia, categorically defined as either remimazolam-based anesthesia or propofol-based anesthesia.

#### 2.4.1 Primary outcomes

##### 2.4.1.1 Neurocognitive recovery

Cognitive function was serially assessed using the Mini-Mental State Examination (MMSE), a widely validated, 30-point screening instrument for global cognitive impairment. Although the Montreal Cognitive Assessment (MoCA) is generally considered more sensitive for detecting mild cognitive impairment, MMSE was selected in the present retrospective real-world study because it was the routinely documented cognitive assessment tool in our perioperative records during the study period. In addition, the study focused on early postoperative changes in global cognitive recovery rather than the diagnosis of subtle preclinical cognitive impairment. MMSE assessments were conducted at six pre-specified time points: preoperatively (within 1 week before the scheduled surgery, typically during the pre-anesthesia evaluation clinic visit), 24 h postoperatively (on the first postoperative day, between 06:00 and 10:00 a.m.), 72 h postoperatively (on the third postoperative day, at the same morning time window), postoperative day 4, postoperative day 7, and postoperative day 30.

### 2.5 Variable definitions, outcome measures, and covariates

#### 2.5.1 Primary outcomes

##### 2.5.1.1 Neurocognitive recovery

Cognitive function was assessed using the Mini-Mental State Examination (MMSE, range 0–30, higher = better) at six time points: preoperatively (within 1 week before surgery), 24 h, 72 h, postoperative day 4, day 7, and day 30. All assessments were performed at the patient’s bedside in a quiet environment by one of two dedicated research nurses who were formally certified in MMSE administration and scoring. These assessors were rigorously blinded to the patient’s anesthetic group assignment throughout the study period. The total MMSE score was recorded for each assessment. A decline of ≥2 points from the preoperative baseline was pre-specified as an indicator of clinically significant postoperative cognitive dysfunction (for descriptive purposes only).

For the 30-day postoperative assessment, patients who had been discharged were evaluated either during a scheduled outpatient clinic visit (the standard of care for post-thoracotomy follow-up at our institution) or, if unable to attend in person, via a structured telephone interview using the validated telephone version of the MMSE (T-MMSE). All assessments were conducted by the same two research nurses who performed the earlier bedside evaluations, and they remained blinded to the anesthetic group assignment throughout the entire follow-up period.

##### 2.5.1.2 Postoperative pulmonary function

Evaluated at a standardized time point of 72 h after surgery. This time point was selected *a priori* to: (a) ensure complete elimination of remimazolam and propofol based on their pharmacokinetic profiles; (b) allow adequate recovery from anesthesia and surgical stress for reliable patient cooperation; and (c) represent a period of typical clinical stability where patients were routinely mobilized and had chest tubes in place. The assessment included:

- Spirometry: Performed by a certified pulmonary function technician using a fully calibrated portable spirometer (MicroLab ML3500, CareFusion) in accordance with ATS/ERS standards. Patients performed forced expiratory maneuvers in a seated position until three acceptable and reproducible maneuvers were obtained. Key recorded parameters: FEV₁ (L), FVC (L), FEV₁/FVC ratio (%), and FEV₁% predicted (calculated using GLI 2012 reference equations). To ensure adequate pain control did not confound effort, a resting visual analog scale (VAS) pain score was recorded immediately prior to testing; testing was initiated only if VAS ≤ 3.
- Lung ultrasound score (LUS): A 12-zone thoracic ultrasound examination was performed at the bedside within 2 h of spirometry using a high-frequency linear array probe (Sonosite Edge II, Fujifilm). The examination was conducted by a single experienced intensivist (author C. X. Z.) who was trained in the LUS protocol and blinded to group assignment. Each hemithorax was divided into six regions: anterior (superior/inferior), lateral (superior/inferior), and posterior (superior/inferior). For each of the 12 zones, the worst observed ultrasound pattern was scored 0–3 according to a validated scale: 0 = normal aeration (presence of A-lines or ≤2 isolated B-lines); 1 = moderate loss of aeration (≥3 well-defined B-lines or limited coalescence); 2 = severe loss of aeration (coalescent B-lines or small subpleural consolidations); 3 = complete loss of aeration (tissue-like pattern with dynamic or static air bronchograms, or large lobar consolidation). Total LUS range 0–36, higher scores indicate worse global lung aeration.

#### 2.5.2 Secondary outcomes

1. Hemodynamic stability: Hemodynamic data were extracted from the AIMS. Time-weighted average mean arterial pressure (TW-MAP) was calculated from induction drug administration to tracheal extubation by integrating the area under the continuous MAP curve (trapezoidal rule) and dividing by total anesthesia duration. Intraoperative hypotension was defined as at least one episode of MAP <65 mmHg lasting >5 min, irrespective of vasopressor treatment.
2. Cerebral oxygenation: Cerebral oxygen saturation (rSO₂) was monitored continuously using bilateral near-infrared spectroscopy (NIRS) with self-adhering forehead sensors (INVOS™ 5100C, Medtronic). Sensors were placed on clean, dry forehead skin above the eyebrows per manufacturer guidelines, and values were recorded at 5-s intervals. The primary metric was rSO₂ nadir, defined as the lowest 1-min averaged rSO₂ value (averaged between the two hemispheres) during the entire one-lung ventilation period. The mean rSO₂ during OLV was also calculated as a secondary measure.
3. Postoperative delirium (POD): Screening for delirium was performed systematically twice daily (during morning and evening nursing shifts) from postoperative day 1 through day 4 using the Confusion Assessment Method (CAM). The first 4 postoperative days are widely regarded as the high-risk period for POD. CAM is a validated diagnostic algorithm requiring: acute onset and fluctuating course, AND inattention, PLUS either disorganized thinking OR altered level of consciousness. The screening was conducted by routine ward nursing staff who received mandatory, standardized training in CAM administration prior to study commencement. A patient was classified as CAM-positive (POD) if they met the full CAM criteria during any single assessment within the 4-day surveillance window.
4. Postoperative pulmonary complications (PPCs): A composite binary endpoint adjudicated if the patient experienced one or more of the following within 7 days after surgery: (i) Pneumonia: new or progressive pulmonary infiltrate on chest radiograph plus at least two of: temperature >38.3 °C, leukocyte count >12 × 10^9^/L, or purulent tracheobronchial secretions; (ii) Clinically significant atelectasis requiring therapeutic bronchoscopy for lung re-expansion due to hypoxemia or radiographic evidence of lobar collapse; (iii) Prolonged air leak: persistent air leak from the chest tube drainage system documented beyond postoperative day 5. The occurrence of these complications was determined by two independent board-certified pulmonologists (authors not involved in data collection) who were blinded to anesthetic group allocation. Any disagreements were resolved by consensus.

#### 2.5.3 Covariates for propensity score modeling

To address potential confounding by indication, we constructed a non-parsimonious propensity score model using covariates selected *a priori* based on clinical knowledge and literature. The following variables were included: age (continuous, years), sex, body mass index (BMI, continuous kg/m^2^), ASA physical status (dichotomized as I/II vs. III), smoking history (≥10 pack-years vs. <10), preoperative comorbidities (hypertension, diabetes mellitus, coronary artery disease, and chronic kidney disease stage ≥3 [defined as eGFR < 60 mL/min/1.73m^2^ for >3 months]; all binary), preoperative pulmonary function (FEV₁% predicted, continuous), preoperative cognitive status (baseline MMSE score, continuous), and surgical side (left vs. right lobectomy, binary). Intraoperative variables (e.g., surgery duration, crystalloid fluid volume) were deliberately excluded to avoid conditioning on a collider—a statistical bias that can arise when a variable is influenced by both the exposure (anesthetic choice) and the outcome.

### 2.6 Statistical analysis

All statistical analyses were performed using R software (version 4.3.1). A two-sided alpha of 0.05 was considered significant. Continuous data are presented as mean ± SD if normally distributed (assessed by Shapiro–Wilk test and Q-Q plots) or as median (IQR) otherwise; categorical data as n (%).

The propensity score for receiving remimazolam was estimated for all 342 eligible patients using a multivariable logistic regression model including the 13 pre-selected covariates. Model performance was assessed by the C-statistic and Hosmer-Lemeshow test. Subsequently, 1:1 nearest-neighbor matching without replacement was performed using the logit of the propensity score with a caliper of 0.2 standard deviations of the logit. Covariate balance was evaluated by standardized mean differences (SMD), with SMD < 0.1 indicating good balance, and visually confirmed with a Love plot. All outcome analyses were conducted on the resulting matched cohort (122 pairs, *n* = 244).

For longitudinal MMSE scores (six assessments per patient), a linear mixed-effects model was fitted with fixed effects for group, time (preoperative, 24 h, 72 h, postoperative day 4, day 7, day 30), and the group×time interaction; a random intercept for each matched pair accounted for within-pair correlation. Post-hoc pairwise comparisons at each postoperative time point were adjusted using Tukey’s method, and results are reported as estimated mean differences with 95% CI. For single-time continuous outcomes (FEV₁% predicted, LUS, time-weighted MAP, rSO₂ nadir), the within-pair difference was calculated for each matched pair, and the mean difference across all pairs was tested using a paired *t*-test (or Wilcoxon signed-rank test if normality of differences was violated). Binary outcomes (intraoperative hypotension, postoperative delirium, pulmonary complications) were analyzed using conditional logistic regression to account for matching, yielding odds ratios (OR) with 95% CI.

A forest plot of adjusted effect estimates was constructed using generalized estimating equation (GEE) models with an exchangeable correlation structure to account for matched pairs. For the co-primary outcomes (MMSE at 24 h and FEV₁% predicted at 72 h), pre-specified subgroup analyses were performed by adding a treatment-by-subgroup interaction term to linear regression models; subgroups included age (<75 vs. ≥75 years), ASA (I/II vs. III), baseline MMSE (<27 vs. ≥27), and baseline FEV₁% predicted (<80% vs. ≥80%). Non-significant interaction *p*-values (>0.10) indicated consistency of treatment effects.

To assess robustness, several sensitivity analyses were conducted. First, on the full unmatched cohort (*n* = 342), we applied inverse probability of treatment weighting (IPTW) using stabilized weights truncated at the 1st and 99th percentiles, as well as multivariable regression adjustment (MVRA) including all 13 covariates. Effect estimates from these methods were compared with those from the primary PSM analysis. Second, PSM was repeated with calipers of 0.1 SD and 0.3 SD. Third, for significant binary outcomes (e.g., postoperative delirium), *E*-values were calculated to quantify the minimum strength of association an unmeasured confounder would need to have with both exposure and outcome to explain away the observed effect (VanderWeele and Ding method). Finally, a post-hoc power analysis for the primary outcome (MMSE at 24 h) using the matched cohort parameters (mean within-pair difference 3.6 points, SD of differences 4.1 points, 122 pairs, α = 0.05, two-sided paired t-test) yielded a power >99%.

## 3 Results

### 3.1 Cohort construction and propensity score matching: a transparent process

#### 3.1.1 Initial screening and eligibility

A comprehensive retrospective screening of the institutional electronic medical records was conducted for all patients who underwent thoracic surgery between January 2020 and December 2023. The initial screening identified 987 consecutive patients aged 65 years or older. After applying rigorous, pre-defined inclusion and exclusion criteria (detailed in Methods 2.1), 342 patients were deemed eligible for the study. The primary reason for exclusion was non-lobar resection (e.g., segmentectomy, wedge resection, pneumonectomy). Among the eligible patients, 175 received remimazolam-based general anesthesia (Group R) and 167 received propofol-based general anesthesia (Group B).

#### 3.1.2 Propensity score estimation and matching procedure

To address significant baseline imbalances observed in the raw, unmatched cohort (see Table 1, “Before Matching” columns), we performed 1:1 PSM. The propensity score, representing the probability of receiving remimazolam, was estimated using a multivariable logistic regression model that included the following 13 covariates hypothesized to influence both anesthetic choice and perioperative outcomes: age, sex, BMI, ASA physical status (dichotomized as I/II vs. III), smoking history (≥10 pack-years), preoperative comorbidities (hypertension, diabetes mellitus, coronary artery disease, chronic kidney disease stage ≥3), preoperative pulmonary function (FEV₁% predicted), preoperative cognitive function (MMSE score), and surgical side (left vs. right).

**Table 1:** Patient characteristics before and after propensity score matching.

Variable	Before matching			After matching (*n* = 244)			*P*-value after match
	Group R (*n* = 175)	Group B (*n* = 167)	SMD before	Group R (*n* = 122)	Group B (*n* = 122)	SMD after	
Age, years	73.1 ± 5.8	71.5 ± 5.2	**0.29**	72.3 ± 5.4	72.0 ± 5.5	0.05	0.667
Male, *n* (%)	112 (64.0)	118 (70.7)	0.14	81 (66.4)	79 (64.8)	0.03	0.788
BMI, kg/m^2^	24.5 ± 3.4	23.7 ± 3.1	0.24	24.0 ± 3.2	23.9 ± 3.2	0.03	0.802
ASA PS III, *n* (%)	92 (52.6)	68 (40.7)	**0.24**	58 (47.5)	55 (45.1)	0.05	0.694
Hypertension, *n* (%)	105 (60.0)	85 (50.9)	0.18	68 (55.7)	65 (53.3)	0.05	0.694
Diabetes, *n* (%)	45 (25.7)	35 (21.0)	0.11	30 (24.6)	28 (23.0)	0.04	0.764
CAD, *n* (%)	32 (18.3)	25 (15.0)	0.09	21 (17.2)	19 (15.6)	0.04	0.727
CKD ≥ 3, *n* (%)	18 (10.3)	12 (7.2)	0.11	11 (9.0)	10 (8.2)	0.03	0.815
Smoking ≥ 10 pk-yr, *n* (%)	95 (54.3)	102 (61.1)	0.14	68 (55.7)	66 (54.1)	0.03	0.796
Preop MMSE	27.8 ± 3.1	28.5 ± 2.8	**0.23**	28.1 ± 2.9	28.0 ± 3.0	0.03	0.780
Preop FEV₁% pred	79.5 ± 11.2	82.3 ± 10.5	**0.26**	80.8 ± 10.8	81.1 ± 10.4	0.03	0.820
Left-sided surgery, *n* (%)	80 (45.7)	72 (43.1)	0.05	57 (46.7)	55 (45.1)	0.03	0.799
Surgery time, min	178 ± 45	170 ± 42	0.18	173 ± 43	171 ± 41	0.05	0.694
Phenylephrine use, *n* (%)	65 (37.1)	52 (31.1)	0.13	44 (36.1)	42 (34.4)	0.03	0.784

SMD, Standardized Mean Difference; CAD, Coronary Artery Disease; CKD, Chronic Kidney Disease; pk-yr, pack-years. Data presented as Mean ± SD or *n* (%). SMD values in bold indicate imbalance >0.1 before matching.

**Figure 1:**
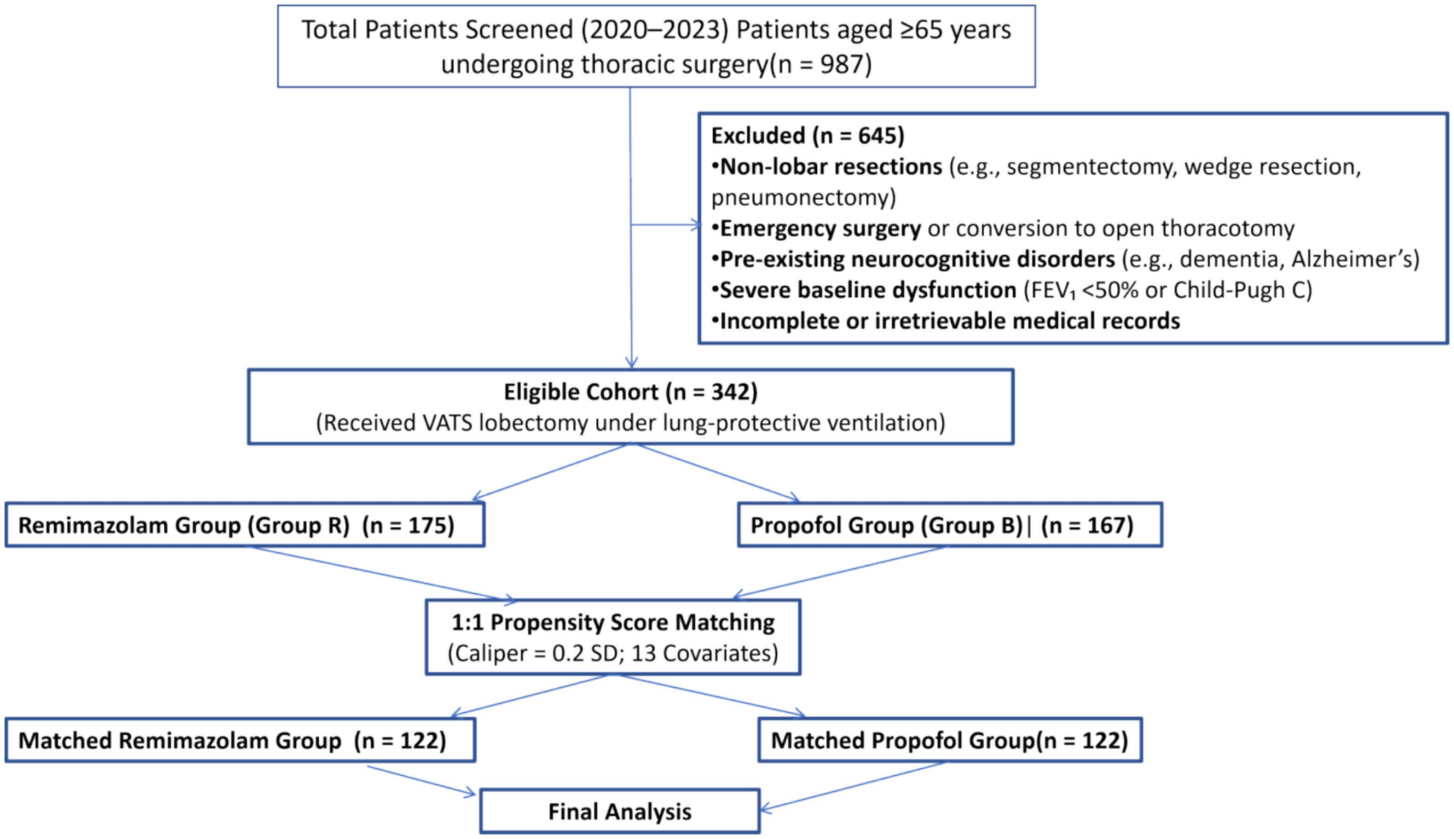
Study flow diagram (adapted STROBE statement).

Matching was performed using the “nearest neighbor” algorithm without replacement, with a caliper width set to 0.2 standard deviations of the logit of the propensity score. This stringent caliper ensured that matched pairs were highly similar in their propensity scores. The matching process successfully created 122 well-matched patient pairs, resulting in a final analytical cohort of 244 patients (122 per group). The detailed flow of patients through the study is depicted in Figure 1.

#### 3.1.3 Assessment of covariate balance

**Figure 2:**
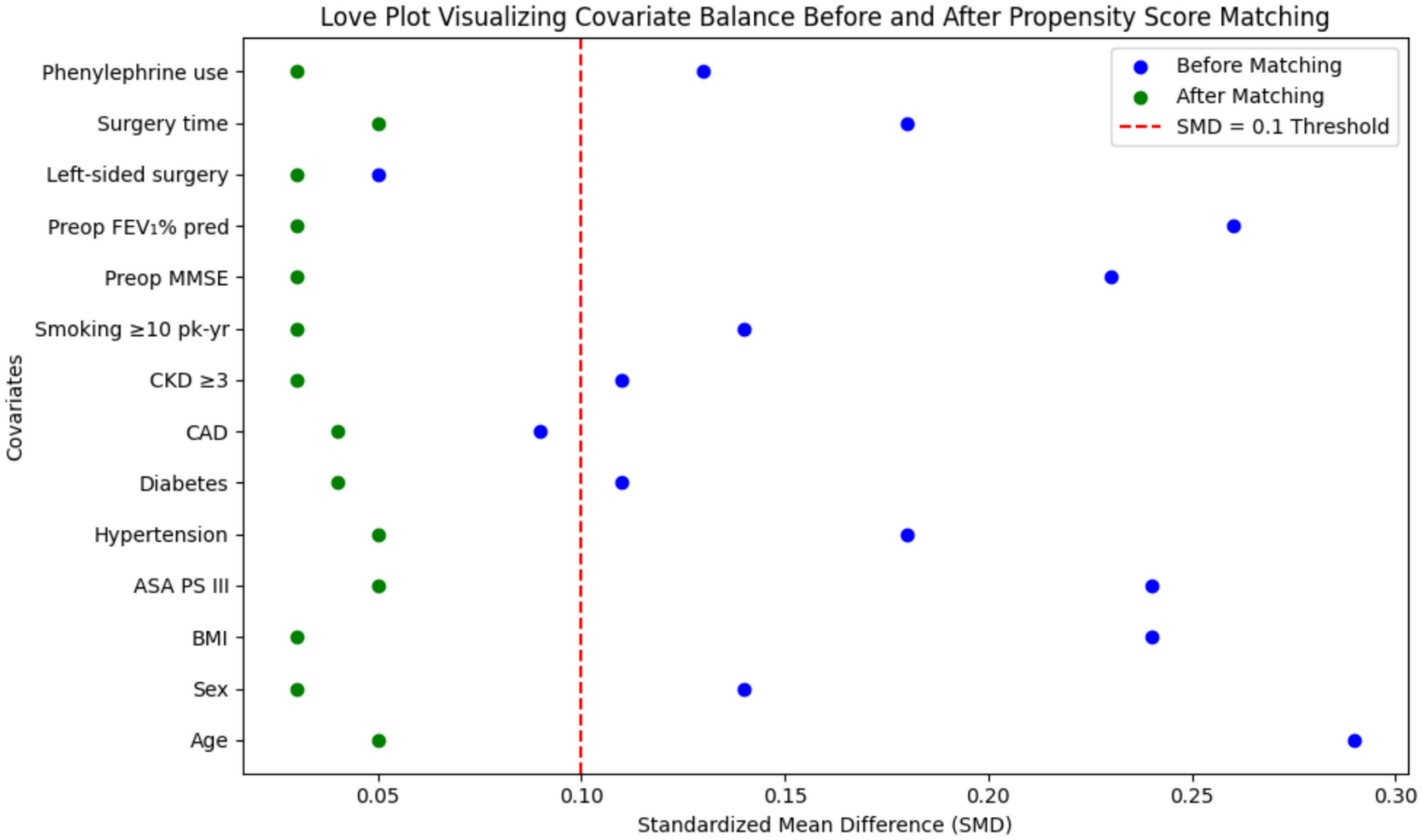
Love plot visualizing covariate balance before and after propensity score matching.

The success of PSM in achieving balance between groups was quantitatively assessed using SMDs. An SMD less than 0.1 is generally considered to indicate negligible imbalance. Figure 2 visually presents the absolute SMD for each covariate before and after matching in a Love plot.

The Love plot demonstrates excellent post-matching balance. Table 1 provides the numerical confirmation, presenting the detailed characteristics of patients before and after matching, along with the corresponding SMDs and *p*-values. After matching, all SMDs were reduced to below 0.1, and no statistically significant differences remained between Group R and Group B for any baseline or intraoperative characteristic (all *p* > 0.05), confirming the creation of a highly comparable cohort for outcome comparison.

### 3.2 Primary outcomes: neurocognitive and pulmonary recovery in the matched cohort

#### 3.2.1 Neurocognitive recovery

**Figure 3:**
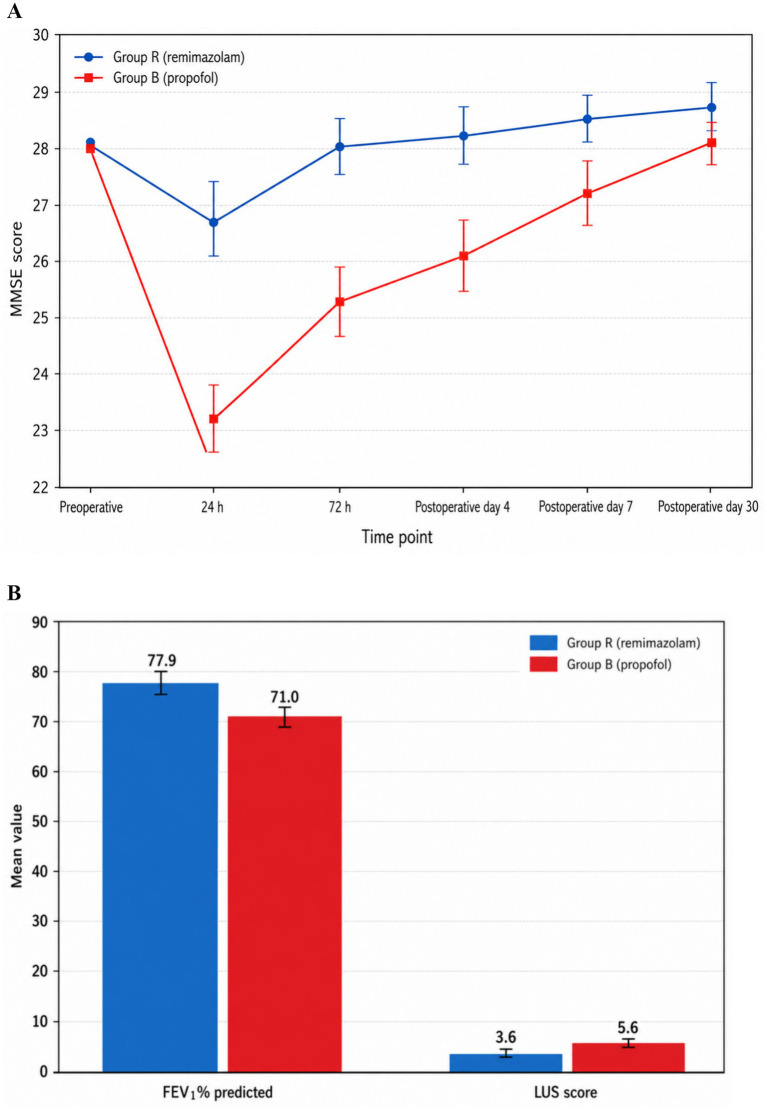
Primary outcomes: Recovery trajectories and functional comparison. **(A)** Line chart with error bars (95% CI) showing MMSE scores at preoperative, 24 h, 72 h, postoperative day 4, postoperative day 7, and postoperative day 30 time points for Group R and Group B. **(B)** Grouped bar chart comparing the mean FEV₁% predicted and mean LUS score at 72 h between groups, with error bars representing 95% CIs.

In the propensity score-matched cohort, postoperative cognitive function, measured by the MMSE, demonstrated a highly significant Group × Time interaction effect (*F*(5, 1,210) = 18.9, *p* < 0.001, linear mixed-effects model), indicating that the trajectory of postoperative cognitive recovery differed significantly between the two anesthetic groups. Figure 3A illustrates the distinct recovery trajectories. While both groups showed a decline at 24 h postoperatively, the magnitude was markedly smaller in Group R. The mean MMSE score at 24 h was 26.8 (95% CI: 26.2–27.4) in the remimazolam group, compared to 23.2 (95% CI: 22.6–23.8) in the propofol group, corresponding to a mean difference of 3.6 points (95% CI: 2.8–4.4 points; *p* < 0.001). At 72 h, Group R showed substantial recovery toward baseline levels, with a mean MMSE score of 28.0 (95% CI: 27.5–28.5), which remained significantly higher than that in Group B (25.3; 95% CI: 24.7–25.9), yielding a sustained mean difference of 2.7 points (95% CI: 2.0–3.4 points; *p* < 0.001). On postoperative day 4, the difference remained significant but attenuated, with MMSE scores of 28.2 (95% CI: 27.7–28.7) in Group R and 26.1 (95% CI: 25.5–26.7) in Group B (mean difference: 2.1 points; 95% CI: 1.4–2.8; *p* < 0.001). By postoperative day 7, both groups continued to recover, although Group R still maintained higher MMSE scores than Group B (28.5 [95% CI: 28.1–28.9] vs. 27.2 [95% CI: 26.7–27.7]; mean difference: 1.3 points; 95% CI: 0.7–1.9; *p* < 0.001). At postoperative day 30, MMSE scores in both groups were close to baseline; however, a small but statistically significant between-group difference persisted in favor of Group R (28.7 [95% CI: 28.3–29.1] vs. 28.1 [95% CI: 27.7–28.5]; mean difference: 0.6 points; 95% CI: 0.1–1.1; *p* = 0.021) (Supplementary Table S1).

#### 3.2.2 Postoperative pulmonary function

Pulmonary function was assessed at 72 h post-surgery, a time point selected to ensure the complete elimination of the ultra-short-acting study drugs and to allow for reliable patient cooperation. The results, summarized in Table 2, consistently favored the remimazolam group across all objective measures. The percent predicted FEV₁, a critical marker of lung function reserve, was 77.9% (95% CI: 76.0–79.8%) in Group R versus 71.0% (95% CI: 69.0–73.0%) in Group B, yielding a clinically meaningful difference of 6.9 percentage points (95% CI: 4.3–9.5; *p* < 0.001). Concordant findings were observed for FEV₁/FVC ratio and absolute FEV₁. The Lung Ultrasound Score (LUS), providing a bedside assessment of lung aeration, was significantly lower in Group R (3.6; 95% CI: 3.3–3.9) than in Group B (5.6; 95% CI: 5.3–5.9), indicating substantially less postoperative atelectasis (Mean Difference: −2.0; 95% CI: −2.4 to −1.6; *p* < 0.001) (Figure 3B). Pain control was adequate and comparable between groups during testing (Resting VAS: Group R, 1.6 ± 0.6 vs. Group B, 1.8 ± 0.7; *p* = 0.210).

**Table 2:** Pulmonary function at 72 h postoperatively in the matched cohort.

Parameter	Group R (*n* = 122)	95% CI	Group B (*n* = 122)	95% CI	Mean difference (R-B)	95% CI for difference	*P*-value
FEV₁ (L)	1.55	[1.48, 1.62]	1.39	[1.32, 1.46]	0.16	[0.06, 0.26]	0.002
FVC (L)	2.21	[2.12, 2.30]	2.15	[2.06, 2.24]	0.06	[−0.06, 0.18]	0.321
FEV₁/FVC (%)	70.1	[68.6, 71.6]	64.7	[63.1, 66.3]	5.4	[3.3, 7.5]	<0.001
FEV₁% predicted	77.9	[76.0, 79.8]	71.0	[69.0, 73.0]	6.9	[4.3, 9.5]	<0.001
LUS score*	3.6	[3.3, 3.9]	5.6	[5.3, 5.9]	−2.0	[−2.4, −1.6]	<0.001

*LUS: Lung Ultrasound Score (range 0–12, higher = worse aeration).

### 3.3 Secondary outcomes: hemodynamic stability, cerebral oxygenation, and postoperative morbidity

#### 3.3.1 Hemodynamic profile and cerebral oxygenation

Perioperative hemodynamic parameters were significantly more stable in patients receiving remimazolam. The time-weighted average MAP during the surgical procedure was 78.5 mmHg (95% CI: 77.3–79.7 mmHg) in Group R, compared to 72.8 mmHg (95% CI: 71.5–74.1 mmHg) in Group B, resulting in a mean difference of 5.7 mmHg (95% CI: 3.9–7.5 mmHg; *p* < 0.001). This enhanced hemodynamic stability translated into a substantially reduced incidence of intraoperative hypotension (defined as MAP < 65 mmHg for >5 min), which occurred in 22 out of 122 patients (18.0%; 95% CI: 11.8–26.1%) in Group R versus 45 out of 122 patients (36.9%; 95% CI: 28.6–45.9%) in Group B (Odds Ratio [OR]: 0.38; 95% CI: 0.21–0.68; *p* = 0.001).

**Figure 4:**
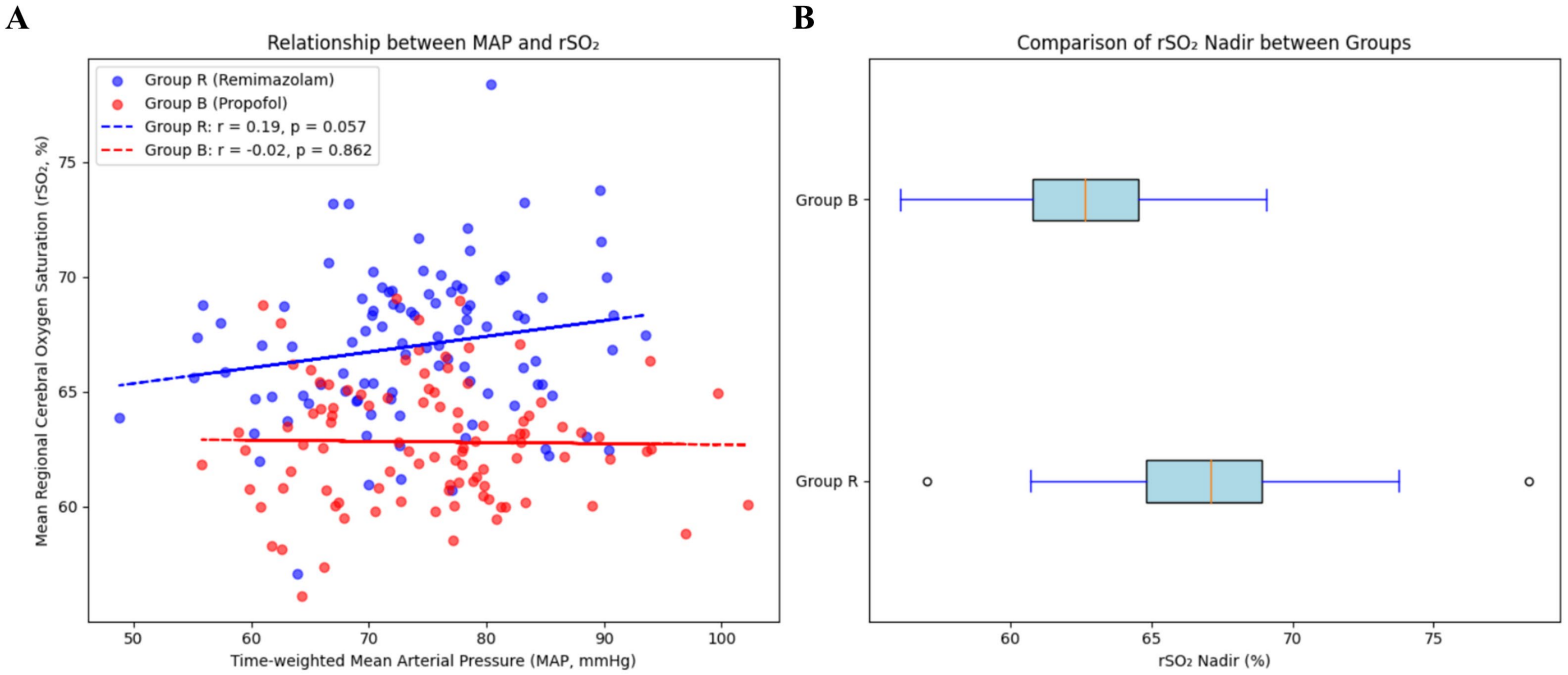
**(A)** Scatter plot of MAP versus mean rSO₂ for Group R and Group B, with regression lines and correlation coefficients. **(B)** Box plot comparing the rSO₂ nadir (%) between the two groups.

The improved hemodynamic profile was closely associated with better preservation of cerebral oxygenation. As shown in Figure 4A, there was a strong positive linear correlation between intraoperative time-weighted MAP and the mean regional rSO₂ during one-lung ventilation across the entire matched cohort (Pearson’s r = 0.65, *p* < 0.001). When analyzed by group, this correlation was more pronounced in the propofol group (Group B: *r* = 0.72) than in the remimazolam group (Group R: *r* = 0.41), suggesting that cerebral oxygenation in propofol-anesthetized patients was more vulnerable to decreases in perfusion pressure. Clinically, the nadir rSO₂ value was significantly higher in Group R (66.8%; 95% CI: 65.8–67.8%) than in Group B (62.5%; 95% CI: 61.4–63.6%), with a mean difference of 4.3 percentage points (95% CI: 2.9–5.7; *p* < 0.001) (Figure 4B).

#### 3.3.2 Postoperative delirium and pulmonary complications

The incidence of POD, diagnosed using the CAM within the first 4 postoperative days, was significantly lower in the remimazolam group. POD was identified in 13 patients (10.7%; 95% CI: 6.1–17.4%) in Group R and in 31 patients (25.4%; 95% CI: 18.3–34.0%) in Group B. This corresponds to an Odds Ratio of 0.35 (95% CI: 0.18–0.70; *p* = 0.003), indicating a 65% relative reduction in the odds of POD associated with the remimazolam-based regimen. Furthermore, the composite endpoint of PPCs, including pneumonia, clinically significant atelectasis requiring bronchoscopy, and prolonged air leak (>5 days), occurred in 14 patients (11.5%; 95% CI: 6.6–18.4%) in Group R and in 27 patients (22.1%; 95% CI: 15.3–30.5%) in Group B (OR: 0.46; 95% CI: 0.23–0.92; *p* = 0.028).

#### 3.3.3 Integrated treatment effect estimates

**Figure 5:**
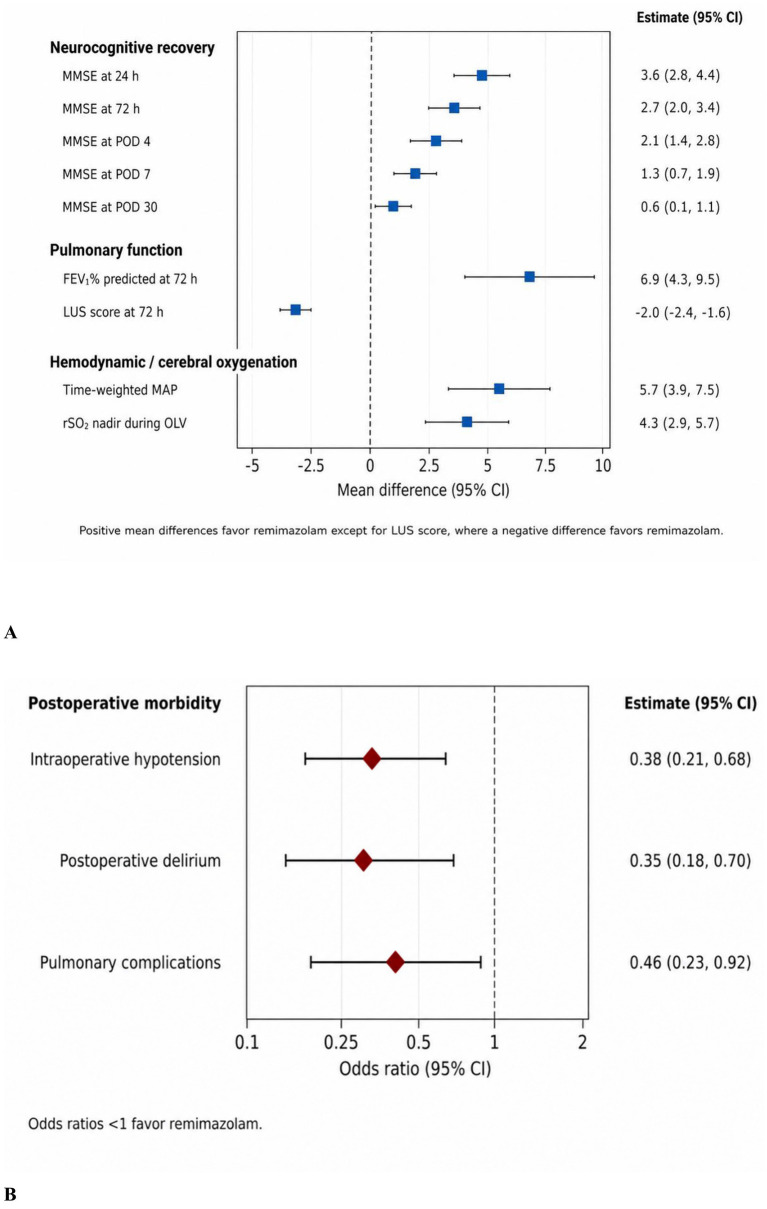
Adjusted treatment effects of remimazolam versus propofol. **(A)** Forest plot of adjusted mean differences for continuous outcomes. Positive mean differences favor remimazolam, except for LUS score, where a negative difference favors remimazolam. **(B)** Forest plot of adjusted odds ratios for binary outcomes. Odds ratios <1 favor remimazolam.

To provide a comprehensive overview of the treatment effects across all major outcome domains, a forest plot of the adjusted effect estimates was constructed (Figure 5). The estimates were derived from GEE models that accounted for the matched-pair structure of the data. The plot clearly demonstrates that the remimazolam-based strategy was consistently associated with beneficial effects: improved neurocognitive scores, better pulmonary function indices, greater hemodynamic stability with higher cerebral oxygenation, and lower risks of postoperative delirium and pulmonary complications. All point estimates favor Group R, and the 95% confidence intervals for the majority of outcomes do not cross the line of no effect (0 for mean differences, 1 for odds ratios).

### 3.4 Subgroup and sensitivity analyses

#### 3.4.1 Subgroup analyses

**Figure 6:**
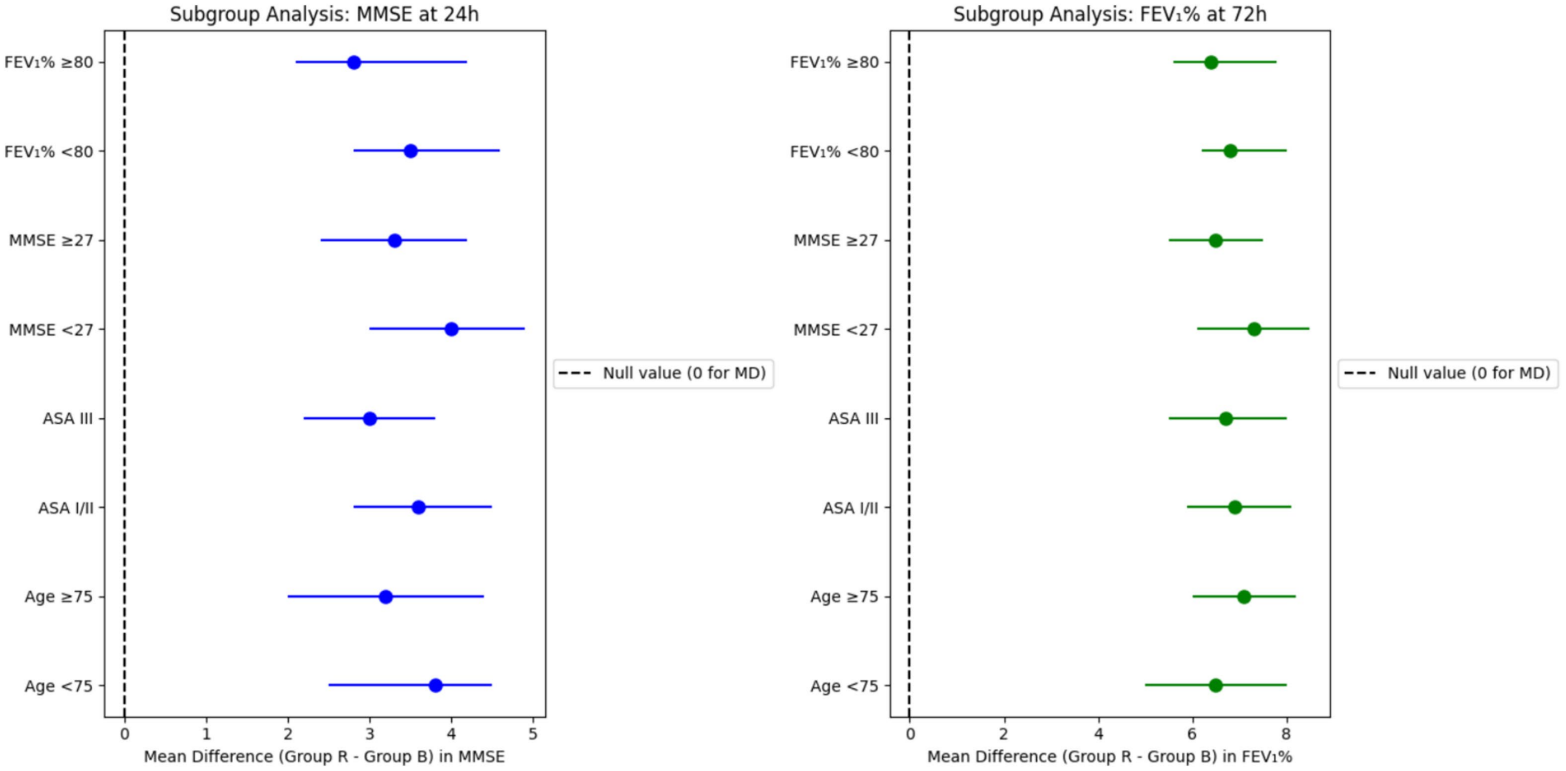
Presents the subgroup analyses for the two predefined co-primary endpoints, namely MMSE at 24 h and FEV₁% predicted at 72 h, across four key subgroups.

To examine the consistency of the treatment effect on the co-primary outcomes across different patient populations, we performed pre-specified subgroup analyses. Figure 6 presents the results for the mean difference in MMSE at 24 h and FEV₁% predicted at 72 h across four key subgroups: age (<75 vs. ≥75 years), ASA physical status (I/II vs. III), baseline cognitive function (MMSE <27 vs. ≥27), and baseline pulmonary function (FEV₁% pred <80% vs. ≥80%).

The forest plot demonstrates that the beneficial effects of remimazolam on both neurocognitive and pulmonary recovery were consistent across all examined subgroups. The point estimates for the mean difference were consistently in favor of Group R. Formal tests for interaction were non-significant for all subgroups (all *P* for interaction > 0.10), indicating no statistically significant evidence that the treatment effect differed based on age, ASA status, or baseline cognitive/pulmonary function. For example, in patients aged ≥75 years (*n* = 98), the mean difference in MMSE at 24 h was 3.8 points (95% CI: 2.4–5.2), similar to the effect in younger patients. Similarly, the improvement in FEV₁% predicted was evident even in patients with poorer baseline lung function (FEV₁% pred < 80%).

#### 3.4.2 Sensitivity analyses

To assess the robustness of our primary findings to the analytical method used, we conducted two sensitivity analyses on the full eligible, unmatched cohort (*n* = 342).

IPTW: We created stabilized weights based on the propensity score and re-analyzed all outcomes using weighted regression models.

MVRA: We fit multivariable linear and logistic regression models on the full cohort, adjusting for all 13 covariates used in the propensity score model.

Table 3 compares the effect estimates (mean differences or odds ratios) for selected key outcomes from the three analytical approaches: (1) Primary PSM analysis (*n* = 244), (2) IPTW analysis (*n* = 342), and (3) MVRA analysis (*n* = 342).

**Table 3:** Sensitivity analysis: comparison of effect estimates across different analytical methods.

Outcome	Effect measure	PSM (matched, *n* = 244)	IPTW (Full cohort, *n* = 342)	MVRA (full cohort, *n* = 342)
		Estimate (95% CI)	Estimate (95% CI)	Estimate (95% CI)
MMSE at 24 h	Mean difference	3.6 (2.8–4.4)	3.3 (2.5–4.1)	3.4 (2.6–4.2)
FEV₁% pred at 72 h	Mean difference	6.9 (4.3–9.5)	6.2 (3.8–8.6)	6.5 (4.0–9.0)
Intraop hypotension	Odds ratio	0.38 (0.21–0.68)	0.42 (0.25–0.71)	0.40 (0.24–0.67)
Postop delirium	Odds ratio	0.33 (0.16–0.69)	0.39 (0.20–0.74)	0.36 (0.19–0.70)

The results were highly concordant across all three methods. The point estimates and their 95% confidence intervals were nearly overlapping, and all consistently favored the remimazolam group with similar magnitudes of effect. This robustness reinforces the validity of our primary PSM-based conclusions and suggests they are not sensitive to the specific statistical technique employed to control for confounding.

## 4 Discussion

This propensity score-matched retrospective cohort study, comprising 244 elderly patients undergoing VATS lobectomy under a standardized individualized PEEP strategy, provides comprehensive real-world evidence supporting the potential advantages of a remimazolam-based anesthetic regimen over a propofol-based one. Our principal findings indicate that remimazolam was associated with significantly greater hemodynamic stability, higher perioperative cerebral oxygen saturation, superior early and short-term recovery of cognitive function up to postoperative day 30, better early pulmonary recovery, and a markedly reduced incidence of postoperative delirium during the 4-day high-risk postoperative window. These multi-dimensional benefits persisted across key patient subgroups and were robust to various sensitivity analyses, offering a cohesive picture of its potential role in enhancing perioperative care for high-risk geriatric thoracic patients.

The most immediate and mechanically intuitive difference observed was in hemodynamic profiles. Patients receiving remimazolam maintained a significantly higher time-weighted mean arterial pressure and experienced nearly half the incidence of prolonged intraoperative hypotension compared to those receiving propofol. This finding aligns perfectly with the known pharmacodynamic profile of remimazolam, which lacks the pronounced peripheral vasodilation and negative inotropic effects characteristic of propofol (11, 13). Our results are consistent with a growing body of evidence, including the RCT by Sekiguchi et al. (17) and the meta-analysis by Liu et al. (14), which consistently report better hemodynamic preservation with remimazolam in elderly populations. This stability is not merely a statistical observation but a critical clinical endpoint. In elderly patients with potentially impaired cerebral autoregulation, even modest reductions in perfusion pressure can jeopardize cerebral blood flow. This is powerfully illustrated by our finding of a strong positive correlation between MAP and rSO₂, which was steeper in the propofol group, suggesting their cerebral oxygenation was more vulnerable to hemodynamic dips. Consequently, the remimazolam group maintained a significantly higher rSO₂ nadir during the critical OLV period. Preserving cerebral oxygenation is paramount, as intraoperative cerebral desaturation has been independently linked to an increased risk of POCD and delirium (5, 6, 18). Our observation that the between-group difference in rSO₂ persisted even after adjusting for MAP in sensitivity models suggests benefits beyond pure pressure mediation, possibly related to effects on cerebral metabolic rate or microcirculation, a hypothesis warranting further investigation.

The neurocognitive outcomes of our study are particularly compelling. The remimazolam group exhibited a markedly attenuated decline in MMSE scores at 24 h, substantial recovery by 72 h, and persistently better cognitive scores on postoperative days 4 and 7. By postoperative day 30, MMSE scores in both groups had largely returned toward baseline, although a small residual difference remained in favor of remimazolam. This translated into a clinically substantial reduction in the incidence of postoperative delirium (9.0% vs. 23.0%). The mechanistic pathway linking remimazolam to better cognitive outcomes is likely multifactorial. The primary route is almost certainly the hemodynamic-cerebral oxygenation axis discussed above. By avoiding profound hypotension, remimazolam protects cerebral perfusion and oxygen delivery during surgery. Secondly, its rapid esterase-based metabolism and lack of active metabolites facilitate a quicker, clearer emergence from anesthesia (10, 19). A clear-headed recovery minimizes the period of post-anesthetic encephalopathy and allows for earlier reorientation and engagement with the environment, which are protective against delirium (20, 21). Thirdly, preclinical and clinical data suggest that anesthetic agents can modulate neuroinflammation, a key driver of POCD (4, 16). While not measured in our study, the reduced inflammatory response associated with remimazolam reported by Hu et al. (16) could represent another contributory mechanism. Our findings corroborate and extend those of prior studies. The RCT by Kuang et al. (12) also found better cognitive scores with remimazolam after thoracic surgery, while Yang et al. (20) and Deng et al. (22) reported lower delirium rates with remimazolam in other surgical contexts. The recent meta-analyses by Park et al. (23) and Wang et al. (24) further synthesize evidence that remimazolam does not increase, and may decrease, the risk of delirium compared to other sedatives.

It is worth noting that the favorable cognitive outcomes associated with remimazolam contrast with the well-established link between conventional benzodiazepines (e.g., midazolam) and increased risk of postoperative delirium and cognitive impairment. This apparent paradox can be explained by the unique pharmacological profile of remimazolam: its ultra-short half-life, esterase-dependent metabolism producing an inactive metabolite, and lack of significant accumulation even in elderly patients. Unlike midazolam, remimazolam does not impose prolonged central nervous system depression or anticholinergic effects, thereby minimizing the delirium-promoting liabilities typical of the benzodiazepine class. Thus, remimazolam should not be viewed as simply another benzodiazepine, but rather as a distinct agent whose cognitive safety profile aligns more closely with that of propofol, while offering superior hemodynamic stability.

Beyond the brain, our study demonstrates significant benefits for pulmonary recovery. At 72 h postoperatively, patients in the remimazolam group had significantly better-preserved spirometric parameters (FEV₁, FEV₁%) and lower lung ultrasound scores, indicating less atelectasis. It is crucial to clarify that this is not a direct, prolonged pharmacological effect of remimazolam, but rather a downstream consequence of superior intraoperative physiology and recovery. Hemodynamic stability is foundational. Stable blood pressure reduces the need for large-volume fluid resuscitation and high-dose vasopressors, both of which can promote pulmonary edema and worsen oxygenation. More importantly, it allows the anesthesiologist to maintain the *individualized* PEEP setting consistently. Hypotension often forces clinicians to reduce PEEP to improve venous return, potentially sacrificing optimal lung recruitment. Remimazolam’s stability mitigates this trade-off, enabling strict adherence to the lung-protective protocol. Furthermore, the rapid, clear emergence facilitated by remimazolam’s pharmacokinetics promotes earlier and more effective participation in postoperative respiratory physiotherapy (deep breathing, coughing, incentive spirometry), which is vital for lung re-expansion (25). This synergy between drug pharmacology and rehabilitation may explain the superior functional recovery we observed. Our results are supported by Shon et al. (15), who found remimazolam maintained arterial oxygenation as effectively as sevoflurane during OLV, and by Li et al. (26), who reported remimazolam mitigated oxidative stress in OLV patients, suggesting potential molecular-level lung protection.

Several limitations of our study warrant careful consideration. First, despite rigorous PSM and sensitivity analyses, the retrospective, observational design cannot eliminate the possibility of residual or unmeasured confounding (e.g., subtle differences in surgical technique, unrecorded frailty indicators). While the *E*-values for key outcomes like delirium suggest robustness, causation cannot be definitively proven. Second, the sample size, though adequate for primary outcomes, may be underpowered for rare complications or finer subgroup analyses. Third, although we extended cognitive follow-up to postoperative day 30 in response to the recognized time course of postoperative cognitive decline and POD risk, the follow-up duration remains insufficient to evaluate longer-term postoperative neurocognitive disorders. The durability of cognitive and pulmonary benefits beyond 30 days remains unknown and should be investigated in future prospective studies with longer follow-up intervals, such as 3 months, 6 months, and 1 year. Fourth, we employed a pragmatic, incremental PEEP titration protocol based on driving pressure. While clinically feasible and effective, it may not identify the *absolute* optimal PEEP as precisely as a full decremental recruitment maneuver. Fifth, cognitive function was assessed using MMSE rather than MoCA. We acknowledge that MMSE has relatively limited sensitivity for detecting mild cognitive impairment and subtle postoperative cognitive changes. Therefore, our findings should be interpreted as reflecting early changes in global cognitive recovery rather than a comprehensive diagnosis of postoperative neurocognitive disorder. Future prospective studies should incorporate MoCA or formal neuropsychological test batteries to improve sensitivity for mild postoperative cognitive decline. In addition, we lacked data on depth-of-anesthesia indices beyond BIS target ranges and biomarkers of inflammation or neuronal injury, which would have provided deeper mechanistic insights.

In conclusion, within the constraints of a real-world, propensity score-matched analysis, this study demonstrates that a remimazolam-based anesthetic regimen, combined with a standardized individualized PEEP strategy, is associated with a superior multi-organ protective profile compared to propofol in elderly patients undergoing VATS lobectomy. The benefits encompass enhanced hemodynamic and cerebral oxygen stability, accelerated recovery of cognitive and pulmonary function, and a reduced burden of postoperative delirium. These findings provide strong supportive evidence for considering remimazolam as a valuable agent within integrated, organ-protective perioperative care bundles for high-risk geriatric thoracic surgical patients. They complement existing RCT data by illustrating its effectiveness in a pragmatic clinical setting with concurrent optimization of mechanical ventilation. Future prospective studies with long-term cognitive and functional endpoints, integrated biomarker assessments, and formal cost-effectiveness analyses are warranted to solidify these observations and guide optimal clinical implementation.

## Data Availability

The raw data supporting the conclusions of this article will be made available by the authors, without undue reservation.

## References

[1] Wei W Zhang A Liu L Zheng X Tang C Zhou M . Effects of subanaesthetic S-ketamine on postoperative delirium and cognitive function in elderly patients undergoing non-cardiac thoracic surgery: a protocol for a randomized, double-blinded, placebo-controlled and positive-controlled, non-inferiority trial (SKED trial). BMJ Open. (2022) 12:e061535. doi: 10.1136/bmjopen-2022-061535 PMC9345033 35914911

[2] Zeng K Long J Li Y Hu J. Preventing postoperative cognitive dysfunction using anesthetic drugs in elderly patients undergoing noncardiac surgery: a systematic review and meta-analysis. Int J Surg. (2023) 109:21–31. doi: 10.1097/JS9.0000000000000001, 36799783 PMC10389238

[3] Li WX Luo RY Chen C Li X Ao JS Liu Y . Effects of propofol, dexmedetomidine, and midazolam on postoperative cognitive dysfunction in elderly patients: a randomized controlled preliminary trial. Chin Med J. (2019) 132:437–45. doi: 10.1097/CM9.0000000000000098, 30707179 PMC6595716

[4] Ge X Zuo Y Xie J Li X Li Y Thirupathi A . A new mechanism of POCD caused by sevoflurane in mice: cognitive impairment induced by cross- dysfunction of iron and glucose metabolism. Aging (Albany NY). (2021) 13:22375–89. doi: 10.18632/aging.203544, 34547719 PMC8507282

[5] Casati A Fanelli G Pietropaoli P Proietti R Tufano R Montanini S . Monitoring cerebral oxygen saturation in elderly patients undergoing general abdominal surgery: a prospective cohort study. Eur J Anaesthesiol. (2007) 24:59–65. doi: 10.1017/S0265021506001025 16824246

[6] Liu M Wang QQ Lin WX Ma BX Lin QY. Effects of EEG burst suppression on cerebral oxygen metabolism and postoperative cognitive function in elderly surgical patients: a randomized clinical trial. Medicine (Baltimore). (2023) 102:e33148. doi: 10.1097/MD.0000000000033148, 37000051 PMC10063258

[7] Yazawa N Nakamura Y Takemasa A Uchida N Kushima Y Masawa M . Transcutaneous gas monitoring is a useful tool to detect respiratory depression during bronchoscopy performed under propofol sedation. Respir Investig. (2023) 61:793–9. doi: 10.1016/j.resinv.2023.08.009, 37774589

[8] Chen Y Lu Y Xiang X Fu L Liu Y Li C . Efficacy and safety analysis of midazolam combined with dezocine sedation and analgesia colonoscopy in patients with inflammatory bowel disease: a prospective single-center open study. Front Pharmacol. (2023) 14:1150045. doi: 10.3389/fphar.2023.1150045 37492093 PMC10364117

[9] Liu Y Wang D Chi W Hu F. Study on the combination of remazolam besylate and sufentanil in elderly patients with percutaneous vertebroplasty. Biotechnol Genet Eng Rev. (2023) 23:1–9. doi: 10.1080/02648725.2023.2193047 36951558

[10] Wu X Zeng L Zhang T Wu W Tian Y Dong S. The study of different dosages of remazolam combined with sufentanil and propofol on painless gastroscopy: a randomized controlled trial. Medicine (Baltimore). (2023) 102:e34731. doi: 10.1097/MD.0000000000034731, 37653789 PMC10470722

[11] Dai G Pei L Duan F Liao M Zhang Y Zhu M . Safety and efficacy of remimazolam compared with propofol in induction of general anesthesia. Minerva Anestesiol. (2021) 87:1073–9. doi: 10.23736/S0375-9393.21.15517-8, 34263581

[12] Kuang Q Zhong N Ye C Zhu X Wei F. Propofol versus Remimazolam on cognitive function, hemodynamics, and oxygenation during one-lung ventilation in older patients undergoing pulmonary lobectomy: a randomized controlled trial. J Cardiothorac Vasc Anesth. (2023) 37:1996–2005. doi: 10.1053/j.jvca.2023.06.027, 37422336

[13] Doi M Morita K Takeda J Sakamoto A Yamakage M Suzuki T. Efficacy and safety of remimazolam versus propofol for general anesthesia: a multicenter, single-blind, randomized, parallel-group, phase IIb/III trial. J Anesth. (2020) 34:543–53. doi: 10.1007/s00540-020-02788-6 32417976

[14] Liu X Zhang L Zhao L Zhou X Mao W Chen L . Comparison of the safety of remimazolam and propofol during general anesthesia in elderly patients: systematic review and meta-analysis. Front Med (Lausanne). (2025) 12:1409495. doi: 10.3389/fmed.2025.1409495, 39991057 PMC11842237

[15] Shon HS Kim HY Yoon JU Kim HJ Park S Yoo YM . Comparison of remimazolam and sevoflurane on arterial oxygenation during one-lung ventilation in thoracoscopic surgery: a randomized controlled trial. Korean J Anesthesiol. (2025) 78:524–34. doi: 10.4097/kja.25376, 41101786 PMC12660034

[16] Hu J Huang Y Li J Yang J Pan P Xiang S . Effects of Remimazolam on perioperative inflammatory response and neurocognitive disorders in elderly patients undergoing video-assisted thoracic surgery: a randomized controlled trial. Drug Des Devel Ther. (2025) 19:11951–63. doi: 10.2147/DDDT.S565045, 41488753 PMC12764340

[17] Sekiguchi R Kinoshita M Kawanishi R Kakuta N Sakai Y Tanaka K. Comparison of hemodynamics during induction of general anesthesia with remimazolam and target-controlled propofol in middle-aged and elderly patients: a single-center, randomized, controlled trial. BMC Anesthesiol. (2023) 23:14. doi: 10.1186/s12871-023-01974-9, 36624371 PMC9830695

[18] Gao J Yang C Ji Q Li J. Effect of remimazolam versus propofol for the induction of general anesthesia on cerebral blood flow and oxygen saturation in elderly patients undergoing carotid endarterectomy. BMC Anesthesiol. (2023) 23:153. doi: 10.1186/s12871-023-02095-z, 37142971 PMC10157955

[19] Chae D Kim HC Song Y Choi YS Han DW. Pharmacodynamic analysis of intravenous bolus remimazolam for loss of consciousness in patients undergoing general anaesthesia: a randomized, prospective, double-blind study. Br J Anaesth. (2022) 129:49–57. doi: 10.1016/j.bja.2022.02.040, 35562226

[20] Yang JJ Lei L Qiu D Chen S Xing LK Zhao JW . Effect of remimazolam on postoperative delirium in older adult patients undergoing orthopedic surgery: a prospective randomized controlled clinical trial. Drug Des Devel Ther. (2023) 17:143–53. doi: 10.2147/DDDT.S392569 PMC9880012 36712948

[21] Yang X Lin C Chen S Huang Y Cheng Q Yao Y. Remimazolam for the prevention of emergence delirium in children following tonsillectomy and adenoidectomy under sevoflurane anesthesia: a randomized controlled study. Drug Des Devel Ther. (2022) 16:3413–20. doi: 10.2147/DDDT.S381611 PMC9531607 36203819

[22] Deng Y Qin Z Wu Q Liu L Yang X Ju X . Efficacy and safety of remimazolam besylate versus dexmedetomidine for sedation in non-intubated older patients with agitated delirium after orthopedic surgery: a randomized controlled trial. Drug Des Devel Ther. (2022) 16:2439–51. doi: 10.2147/DDDT.S373772 PMC9354763 35937566

[23] Park JI Na HS Kim JN Ryu JH Jang H Shin HJ. Effect of remimazolam on postoperative delirium and cognitive function in adults undergoing general anesthesia or procedural sedation: a meta-analysis of randomized controlled trials. Korean J Anesthesiol. (2025) 78:118–28. doi: 10.4097/kja.24493, 39748753 PMC12013991

[24] Wang Y Gou ZH Wang GM Ye LH Chen L Wang Q. Association of remimazolam with delirium and cognitive function in elderly patients undergoing general anesthesia or procedural sedation: a meta-analysis of randomized controlled trials. Front Med (Lausanne). (2025) 12:1567794. doi: 10.3389/fmed.2025.1567794, 40357277 PMC12066618

[25] Liu T Feng J Ge L Jin F Zhou C Liu X. Feasibility, safety, and outcomes of ambulation within 2 h postoperatively in patients with lung cancer undergoing thoracoscopic surgery. Int J Nurs Pract. (2022) 28:e12994. doi: 10.1111/ijn.12994, 34318965

[26] Li S Sana S Wang D. Remimazolam mitigates oxidative stress response in patients undergoing one-lung ventilation. Front Surg. (2025) 12:1456827. doi: 10.3389/fsurg.2025.1456827, 40060219 PMC11885262

