# Supplementary material for: Remimazolam is associated with superior cerebral and pulmonary protection over propofol in elderly thoracic surgery: a real-world study validated by propensity score matching
Source: Front Med (Lausanne). 2026 Jun 3;13:1805497. doi: 10.3389/fmed.2026.1805497 (PMC13272152; doi:10.3389/fmed.2026.1805497)
Supplement: Supplementary file 1 [file Table_1.docx]

Supplementary Table 1. Longitudinal MMSE Scores in the Propensity Score-Matched Cohort

| **Time point** | **Group R (n=122)** | **Group B (n=122)** | **Mean difference** | **95% CI** | **P-value** |
| --- | --- | --- | --- | --- | --- |
| Preoperative | 28.1 | 28 | 0.1 | -0.4 to 0.6 | 0.78 |
| 24 h | 26.8 | 23.2 | 3.6 | 2.8 to 4.4 | <0.001 |
| 72 h | 28 | 25.3 | 2.7 | 2.0 to 3.4 | <0.001 |
| Postoperative day 4 | 28.2 | 26.1 | 2.1 | 1.4 to 2.8 | <0.001 |
| Postoperative day 7 | 28.5 | 27.2 | 1.3 | 0.7 to 1.9 | <0.001 |
| Postoperative day 30 | 28.7 | 28.1 | 0.6 | 0.1 to 1.1 | 0.021 |

Note: Data are presented as estimated marginal means derived from the linear mixed-effects model. CI: confidence interval; MMSE: Mini-Mental State Examination; Group R: remimazolam group; Group B: propofol group.
